# Blood vessel assessment using computed tomography : Effects of ephedrine on uterine artery

**DOI:** 10.3389/fphar.2022.890246

**Published:** 2022-08-23

**Authors:** Yibo Yin, Can Liu, Guangjian Gao, Jingjing Li, Xuechen Long, Peijin Zhang, Wenjun Guo

**Affiliations:** ^1^ Department of Anesthesia, First Affiliated Hospital of Wannan Medical College, Wuhu, China; ^2^ Nuclear Medicine Department, First Affiliated Hospital of Wannan Medical College, Wuhu, China; ^3^ Department of Nosocomial Infection Management, First Affiliated Hospital of Wannan Medical College, Wuhu, China

**Keywords:** computed tomography, vasopressors, pharmacodynamics, uterine artery, animal models

## Abstract

Background: Ephedrine increased blood pressure due to the contractile properties of resistance vessels. Excessive contraction of the uterine arteries might cause fetal distress. This study was to determine the diameter of the uterine artery of female New Zealand rabbits after the administration of different doses of ephedrine using CT.

Methods: Thirty-two rabbits were randomly divided into a control group (Group C), low dosage group (Group L), medium dosage group (Group M) and high dosage group (Group H). Normal saline and doses corresponding to the human dose of 7.5, 15 and 30 mg of ephedrine were injected respectively. The marginal ear and uterine artery diameters were measured 5, 10, 15, 30, and 45 min after injection using CT, and the hemodynamic changes were recorded.

Results: The increase in mean arterial pressure in group M (p = 0.009), and H (p = 0.013) was higher than that in group C. Compared with group C, substantial contraction of the marginal ear artery was observed at the three doses of ephedrine. There were no differences in the uterine artery diameter among groups L, M and C, However, in Group H, a significant contraction of the uterine artery compared with the other groups (p < 0.001) was observed.

Discussion: CT can be used to evaluate the effects of drugs on organs and blood vessels. Ephedrine can not only constrict the peripheral blood vessels but also do not affect the uterine artery at a dose of 15 mg or less. However, the dose should not exceed 30 mg, which may cause severe uterine artery depression.

## Introduction

Spinal anesthesia is the most common method for cesarean section (Saravanan et al., 2006; Kinsella et al., 2018). However, approximately 80% of patients develop hypotension after anesthesia. The hypotension event can affect uteroplacental circulation, and impaired uteroplacental circulation is one of the main causes of severe fetal acidosis and even fetal death (Loughrey et al., 2005; Withers et al., 2009). Previous research has shown that ephedrine is beneficial to maintain uteroplacental circulation and constrict peripheral blood vessels compared with other vasoactive agents (James et al., 1970; McGrath et al., 1994). However, the dose of ephedrine needed to reverse the symptoms of hypotension varies depending on individual sensitivity (Ali Elnabtity Amand Selim, 2018; Hassani et al., 2018). With the increase in dose, the relationship between different blood vessels and ephedrine dose has rarely been reported.

Vasoactive drugs increase blood pressure owing to the contractile properties of resistance vessels. Moreover, changes in the uterine artery may be similar to those in resistance vessels under the action of vasoactive drugs. Many methods, such as umbilical artery pH and Apgar score, are used to indirectly evaluate the effect of ephedrine on the uterine artery. Additionally, Doppler ultrasound is widely used to evaluate hemodynamics, usually by measuring the vascular flow *via* the pulsatility index (PI) or resistance index. However, as the uterine artery is located deep in the abdominal cavity and surrounded by tissue, the measurement of the uterine artery is challenging and the estimation of vessel diameter changes by Doppler ultrasound is not accurate. Thus, a more direct measurement of vessel diameter is needed to quantitatively assess the effect of ephedrine. Computed tomography (CT) has demonstrated excellent penetrating ability, it can clearly show some organs and arteries, offers great potential to explore smaller branches of blood vessels, and can be used to diagnose vascular diseases such as stenosis and occlusion. Furthermore, the three-dimensional reconstruction of CT images can be utilized to accurately measure blood vessel diameter (Mon et al., 2017; Chen et al., 2014). Therefore, in this study, we observed the changes in the uterine and peripheral artery diameters using CT imaging after the injection of different doses of ephedrine.

The primary aim of this study was to explore the feasibility of measuring the uterine and peripheral artery diameter after the administration of different doses of ephedrine using CT. The secondary aim was to determine whether the maintenance effect of ephedrine on the uterine artery changes with the increase in dose. The results of this study will provide a reference for the scientific and rational use of ephedrine in the clinic, and, ultimately, improve the safety of patients and fetuses undergoing cesarean section.

## Materials and methods

### Research subjects

The experimental protocol was approved by the Animal Research Ethics Committee of Wannan Medical College (Approval No. 2019-018), and all procedures were performed in accordance with the United Kingdom Animals (Scientific Procedures) Act of 1986. In total, Thirty-two healthy non-pregnant female New Zealand rabbits (mean weight ± SD: 3.32 ± 0.21 kg; age: 5–6 months) were used in the study. All rabbits were housed in the Laboratory Animal House at a temperature of 22 ± 1°C and humidity of 55 ± 5%, under a 12-h light/dark cycle with free access to water and chow.

### Anesthesia and operation

Under local anesthesia with 1% lidocaine, the left and right marginal ear veins were pierced with an indwelling needle and firmly fixed after heparinization. Anesthesia was induced in every rabbit using sodium pentobarbital (50 mg/kg; XinYu Biotechnology, Shanghai, China) and maintained by continuous infusion of sodium pentobarbital (0.05 mg/kg/min) through an injection pump (LD-P2020 Anesthesia Pump, Lande Medical Ltd., Shanghai, China). The other ear vein was used for the injection of the contract agent (ioversol injection, Hengrui Pharmaceutical Co., Ltd. Nanjing, China). When the corneal reflex and pain reflexes disappeared, the depth of anesthesia met the requirement for surgery. To eliminate the interference of the bladder to the image, urethral catheterization was performed before the experiment. The rabbit was placed on a sterile operating table, the rabbit’s abdomen was sterilized, and surgical towels were spread over the abdomen. A midline laparotomy was performed, and the incision continued to the upper edge of the pubic bone. The intestinal tract, both cervices, and the uterus were observable, as was the scattered vascular structure of the uterus. Then, the uterus was separated from the intestines with a piece of sterile gauze to achieve a clear and undisturbed vascular image. To obtain the hemodynamic changes during the drug cycle, mean arterial pressure was measured using a non-invasive blood pressure measuring device for animals (NIBP220A, Ranzhe Instrument Equipment Co., Ltd., Shanghai, China). The measurement was performed in the middle of the right forelimb radius.

### Angiography imaging

The 32 female rabbits were randomly divided into four groups using a free online randomization tool (http://www.randomizer.org): the control group (Group C), equivalent human dose of 7.5 mg ephedrine (Group L), 15 mg (Group M), and 30 mg (Group H). The purpose of the design of this drug gradient was to observe the difference within the therapeutic dose. The low dose of ephedrine (0.332 mg⋅kg^−1^) used in this study was converted from a human equivalent dose based on body surface area using the following formula from the US Food and Drug Administration: assuming a pregnant woman weight of 70 kg, the pregnant woman equivalent dose was 7.5 mg × 70 kg-1 (0.107 mg⋅kg-1) = 0.107 × 3.1 = a rabbit dose of 0.332 mg⋅kg-1; the conversion coefficient 3.1 was used to account for the difference in body surface area between a rabbit and a human (Wang et al., 2018). The doses used in groups M and H were also calculated in this way.

The contrast agents were injected into the ear veins with a dual chamber power injector (Leibs Industrial Co., ltd., Shanghai, China) at a dosage of 1.5 ml/kg, and rate of 1.5 ml/s. The head to the base of the thigh was quickly scanned using a Definition Flash 128 row dual source X-ray computed tomography machine (SOMATOM Definition Flash; Siemens Medical Solutions, Erlangen, Germany) and recorded (scan parameters: tube voltage 80 kV; tube current 310 mAs; detector width 80 mm; pitch 0.922; lamination thickness 5 mm; interval between layers 5 mm). Rabbits in groups L, M and H were then injected with the respective doses of ephedrine, and Group C was administered normal saline. Based on the blood concentration metabolism of ephedrine, CT scans were performed 5, 10, 15, 30, and 45 min after the administration of ephedrine (Persky et al., 2014). For CT scans, 741 slices were collected. The interval between every two slices was a fixed value of 0.06 mm. The total acquisition time was 39 s.

The original pictures collected from the dynamic CT were transferred to the imaging workstation. A professional processing software (Vitrea Fx6.2.3, Vital Images, Minnetonka, MN, United States) was used to analyze the data on the workstation and calculate the diameters of the uterine and marginal ear arteries (hereinafter referred to as a peripheral artery), and mark them with arrows. The rabbit marginal ear artery (representing the terminal microcirculation) is an important peripheral blood pressure regulator (Harvey and Knowles TandMurison, 2012; Ramos-Alves et al., 2012).

Each measurement was repeated twice, and the average value was recorded. All measurements were performed by a senior professional imaging doctor, who was not informed of the medication status of the rabbits. Addition, to ensure measurement accuracy, the parameters of all machines, concentration of the contrast agent, position of the measurement, and position of the rabbit were fixed. The continuous injection of anesthetics aided the latter two requirements. All rabbits were euthanized by the intravenous injection of a lethal dose of sodium pentobarbital (150 mg/kg) after image acquisition and then when breathing and heartbeat stopped, they were placed in medical waste bags and disposed of by professionals in the central laboratory.

### Statistical analysis

In this study, thirty-two complete datasets were collected and used as the total sample size. The primary outcome measure was the difference in the rate of change of the uterine and peripheral artery diameters among different groups. Quantitative data are presented as mean ± standard deviation (SD). A repeated-measures analysis of variance model was used to examine group differences in measurements over time. The Greenhouse-Geisser procedure was used after checking for variance-covariance matrix sphericity assumptions. Intergroup comparisons of vessel diameter at different time points were analyzed using one-way ANOVA on ranks, using Tukey’s *post-hoc* test for multiple comparisons. The effects of ephedrine infusion on hemodynamic variables was also analyzed using this method. OriginPro 2017 (OriginLab, Northampton, MA, United States) was used to draw graphics. All statistical analyses were performed using SSPS 18.0 (IBM Corporation, Armonk, NY, United States) *p* < 0.05 was considered statistically significant.

## Results

All 32 rabbits provided data for image collection and measurement. The maximum intensity projection and low-density images clearly show the shape of the blood vessels of each organ and that the uterine artery is filled with contrast agent.

**FIGURE 1:**
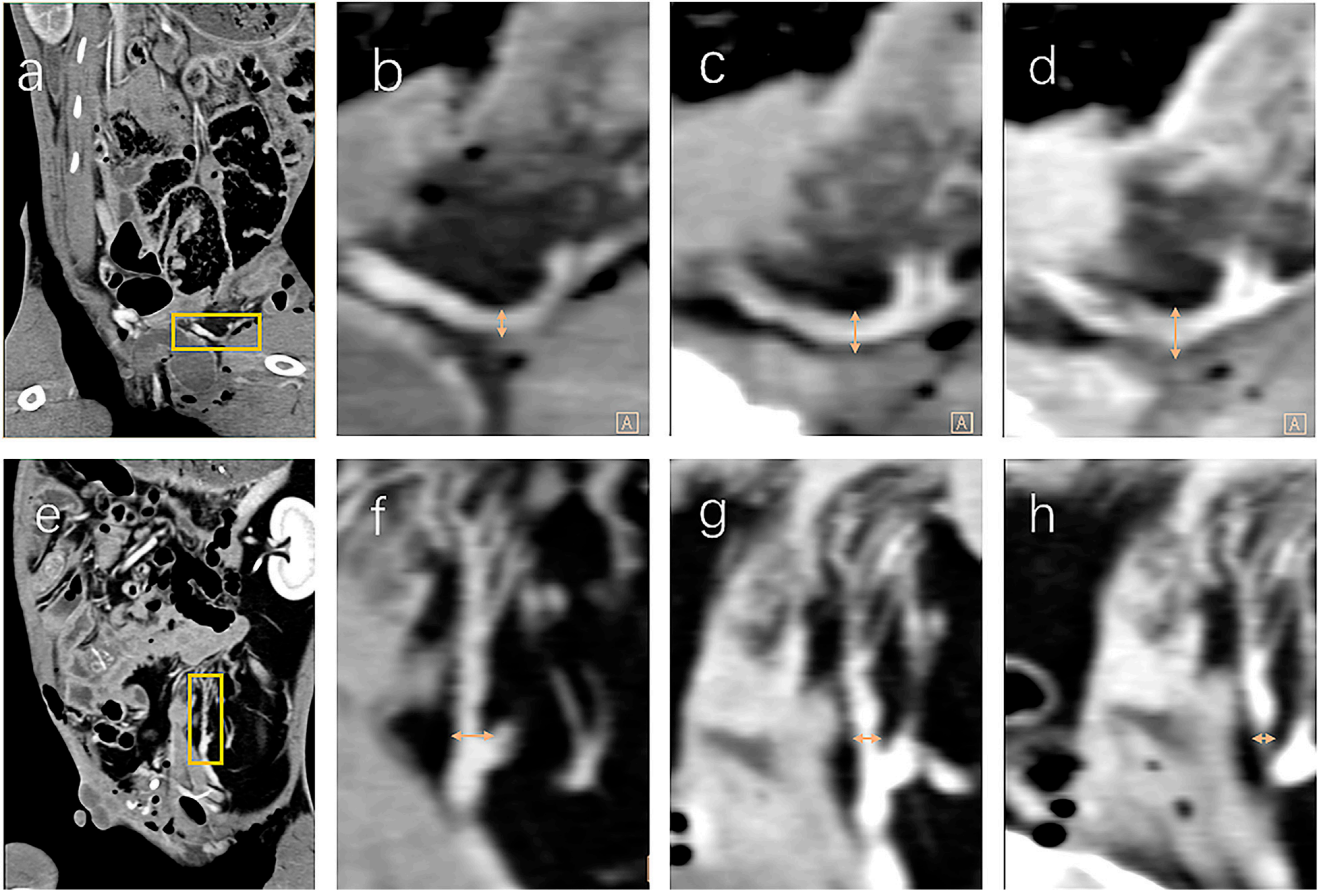
For each row (Figures 1A–H), the figures are from the same rabbits. The computed tomography sagittal plane shows the abdominal cavity of the rabbit and the uterus is marked with a yellow square (Figure 1A). After magnification, the initial distance of the uterine artery (Figure 1B), the blood vessels dilate after 15 min (Figure 1C), until the dilation is obvious at 30 min (Figure 1D). Sagittal plane scan of another rabbit with the uterus marked by the yellow square (Figure 1E). Initial distance of the uterine artery (Figure 1F), vasoconstriction at 15 min after high dose ephedrine (Figure 1G), and final severe contraction at 30 min (Figure 1H). High-density shadows in surrounding organs due to penetration of contrast media, but the blood vessels are severely stenotic, or even transiently interrupted (Figure 1H). The diameter of the uterine artery is marked with a yellow arrow.

The effect of ephedrine on the uterine artery in the different groups is shown in Figure 1; a representative example of a dilated and a contracted uterine artery. Figure 1B shows the diameter of the initial uterine artery without medication. Observe the slight dilation of the uterine artery diameter 5 min after the equivalent 15 mg ephedrine was administered (Figure 1C). This diameter expands to a maximum after 30 min (Figure 1D). Dose of 7.5 mg and 15 mg ephedrine caused an expansion of 5% and 7%, respectively, in the uterine artery diameter after 5min compared to the initial diameter, after which a slow increase was observed, reaching a peak after 45 min with 20% and 34% expansion. After giving the equivalent 30 mg of ephedrine, the typical manifestation of the sequential contraction of the uterine artery over time is shown in Figures 1F–H. The diameter of the uterine artery contracted by 27% in 10 min in this group, and then a gradual recovery was observed.

**FIGURE 2:**
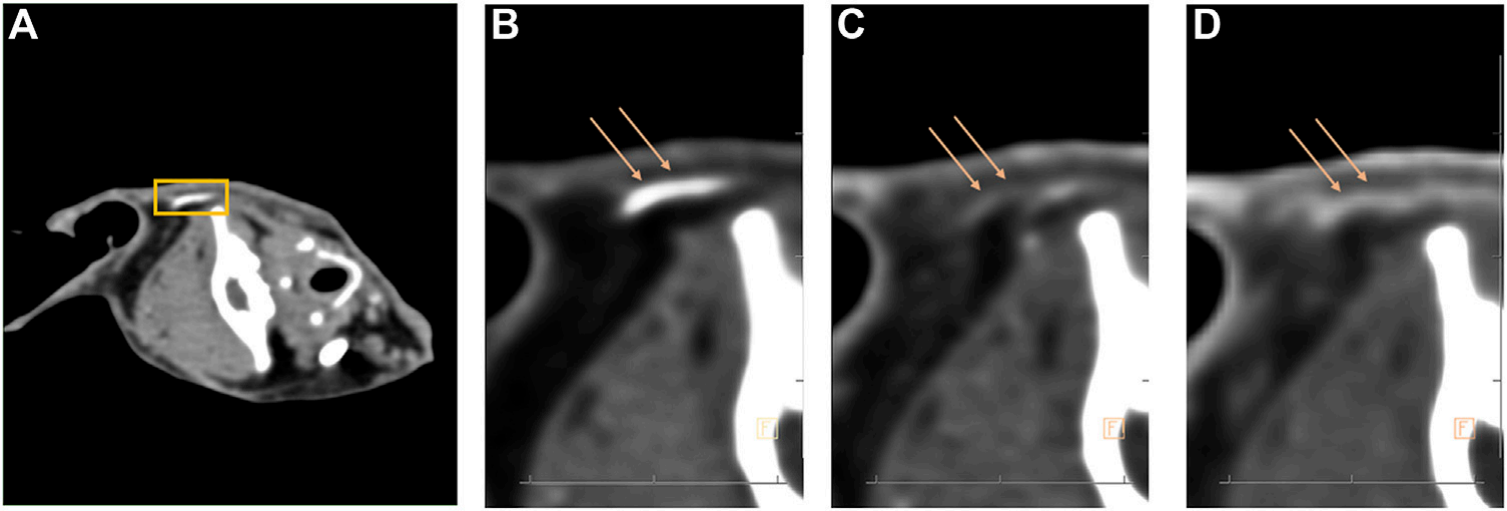
Computed tomography scans the sagittal plane of the entire skull and the marginal ear artery was marked with a yellow square (Figure 2A). The auricular artery is filled with blood vessels (Figure 2B), and the blood vessels are significantly constricted after administration of ephedrine (Figure 2C). The diameter of the marginal auricular artery partially recovered within 30 min (Figure 2D). The loss of visibility of blood vessels due to narrowing of blood vessels and insufficient filling of contrast agent (Figure 2C).

The changes in the marginal ear arteries under the action of ephedrine are shown in Figure 2. Figure 2B is the initial blood vessel image, but after 7.5 mg of ephedrine was administered, the blood vessel contracted substantially, which made the angiography insufficient to fill the blood vessel, and the blood vessel appeared as a shadow and therefore could not be measured (Figure 2C). The same phenomenon was observed in treatment groups. Mean arterial pressure (MAP) is determined by cardiac output and peripheral resistance. Repeated measurement analysis showed that the MAP of groups M and H had substantial changes at different time points. The difference in systolic blood pressure is most obvious between the groups at 10 min, and with the increase in dose, the blood pressure also increased (Table 1).

**TABLE 1:** Hemodynamic parameters of rabbits between groups (mean arterial pressure, MAP, mm Hg, data are expressed as means ± SD, *n* = 30 for each group).

	T_0_	T_5_	T_10_	T_15_	T_30_	T_45_
Group C	85.32 ± 6.75	88.65 ± 4.88	85.85 ± 5.95	88.14 ± 3.93	89.72 ± 5.75	84.69 ± 3.97
Group L	90.25 ± 3.85	92.62 ± 3.59^a^	95.28 ± 3.49^a^ ^,^ ^b^	97.72 ± 3.45^a^ ^,^ ^b^	94.49 ± 6.45	97.49 ± 5.58
Group M	89.14 ± 4.75	111.83 ± 6.95^a^ ^,^ ^b^	113.39 ± 5.89^a^ ^,^ ^b^	117.63 ± 4.52^a^ ^,^ ^b^	109.85 ± 4.39^a^ ^,^ ^b^	102.45 ± 7.58^b^
Group H	91.33 ± 2.18	115.41 ± 3.28^a^ ^,^ ^b^	135.15 ± 3.35^a^ ^,^ ^b^	141.49 ± 6.38^a^ ^,^ ^b^	138.28 ± 5.49^a^ ^,^ ^b^	137.49 ± 3.75^a^ ^,^ ^b^

a *p* < 0.05 compared with the T_0_.

b *p* < 0.05 compared with group c.

**FIGURE 3:**
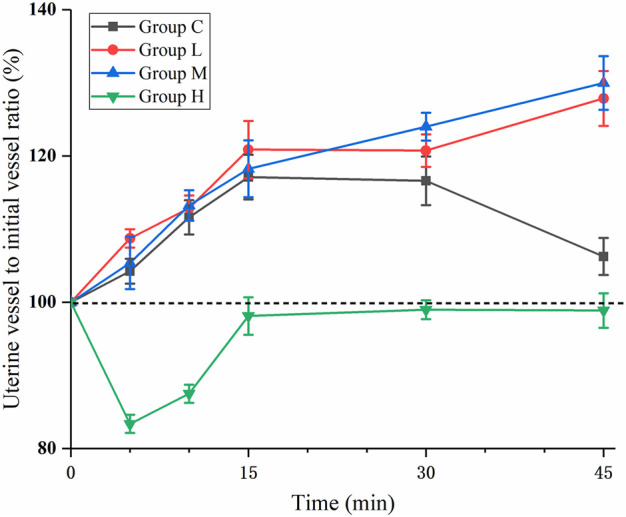
Percentage uterine artery change over time. The value is calculated as the ratio of the diameter of the uterine artery to the initial diameter at each time point. In Group H, the value decreased rapidly and reached its nadir after 5 min (*p* < 0.05 compared with C, L, and M).

**FIGURE 4:**
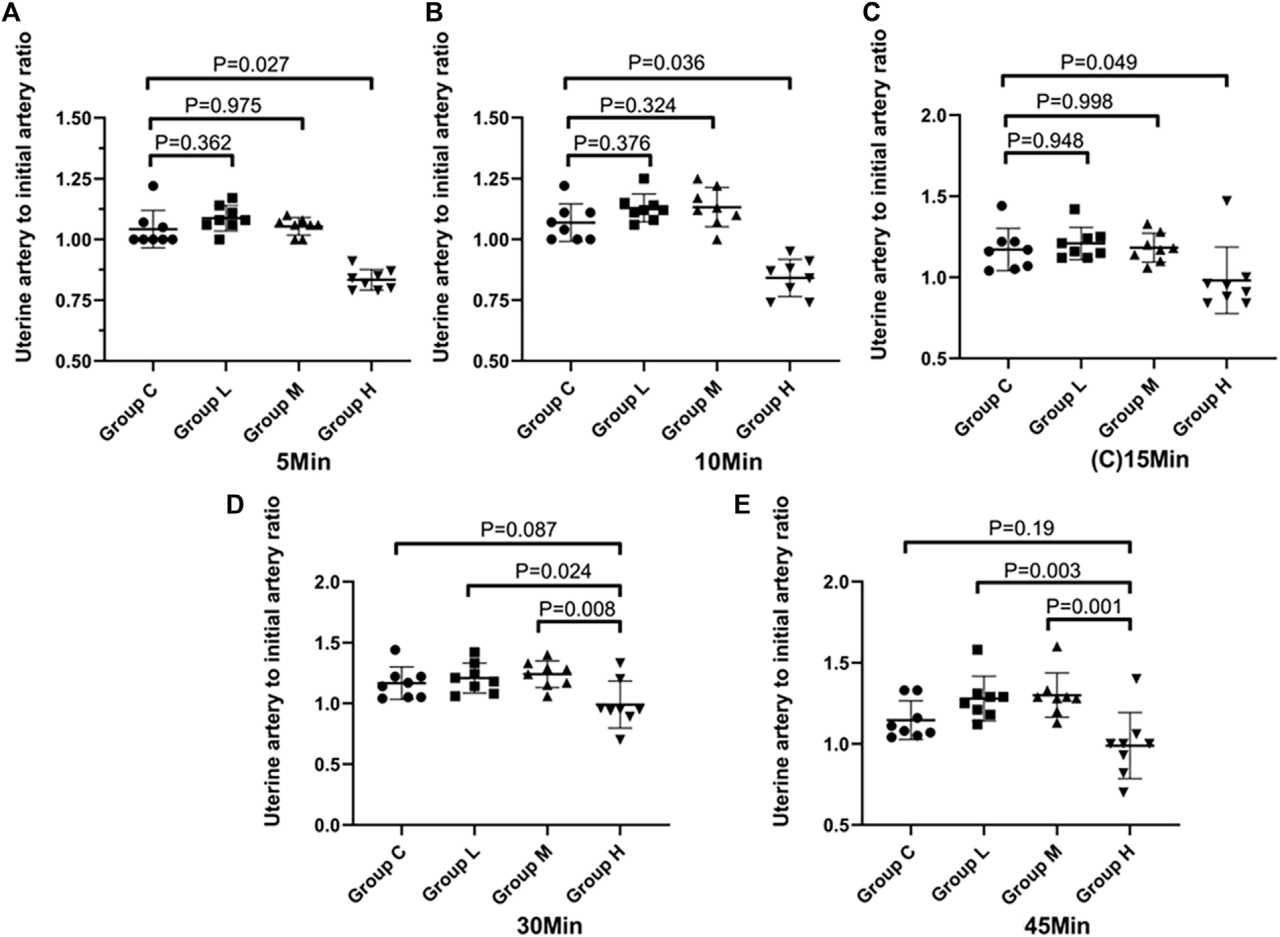
Distribution of the ratio of the diameter of the uterine artery to the initial diameter at different time points. The black line is used to link two groups with statistical significance.

The diameter of the uterine artery was significantly different among groups and over time within each group (*p* < 0.05). Figure 3 shows the uterine artery diameter to initial diameter ratio over time after ephedrine injection. In groups L and M, there was no significant difference in the uterine artery diameter compared with Group C (*p* = 0.82). In Group H, the uterine artery diameter was significantly smaller than that in the other three groups (*p* < 0.001), and the diameter continued to decline within 10 min, indicating that the blood vessels were continuously contracting. The uterine artery diameter to initial diameter ratio of each sample at each time point are presented in Figure 4. After 10 min, the ratio of more than two samples in group L was larger than that in group C. Similar results were observed in Group M. In Group H, the uterine artery diameter was lower than that in the other three groups at each time point, this phenomenon was most significant at 10 min.

## Discussion

In this study, the measurement of CT images showed that all three doses of ephedrine effectively constrict peripheral blood vessels after injection. Moreover, ephedrine does not interfere with the diameter of the uterine artery, or increases the diameter in some cases, at doses of 7.5 and 15 mg. However, in the 30 mg dose group, not only the peripheral artery but also the uterine artery was significantly contracted, and the inhibitory effect lasted until the end of the experiment.

Ephedrine was isolated from an herbal medicine in 1887 and has been widely used ever since. It excites adrenergic α and β receptors directly and indirectly, *via* the promotion of the release of norepinephrine from nerve endings. Additionally, ephedrine increases the contractility of the heart, expands the coronary and intracranial arteries by stimulating β-receptors, and promotes the contraction of skin, mucous membranes, and visceral blood vessels by stimulating α-receptors to increase venous return and blood pressure (Kobayashi et al., 2003; Docherty, 2008). According to previous reports, the main blood vessels supplying the uterus run within the endometrium and smooth muscle and are surrounded by an abundance of adrenergic nerves (Bower, 1966; Brauer, 2008). Some research showed that adrenergic receptors in the uterus are in a state of inhibition at different stages of pregnancy, which suggests that neurons are affected by predisposing factors, such as hormones, body fluids, or drugs (Bulat and Kannan MSandGarfield, 1989; Klukovits et al., 2002). Therefore, we speculate different doses of ephedrine affect adrenergic nerves in different locations, resulting in opposite effects in the uterus.

Similar results were obtained by Ralston et al. using a special flow tube (Ralston and Shnider SMandDeLoorimier, 1974). They measured a decrease in uterine blood flow after the infusion of a high dose of ephedrine. However, an increase in uterine blood flow was not apparent at a normal dose of ephedrine. This subtle difference with our study in the normal dose group may have been caused by the difference in sensitivity between the experimental methods.

The density of blood vessels and extravascular tissue is significantly different after the injection of contrast medium. CT can accurately measure subtle changes in blood vessels, which greatly improves the accuracy of measurement compared with other methods. For example Alahuhta et al. concluded that the pulsatility index (PI) does not change significantly when 5 mg of ephedrine is administered to maintain blood pressure (Alahuhta et al., 1992), whereas Ducros et al. showed that vascular resistance decreases, and flow rate increases at similar doses (Ducros et al., 2002). Although PI is a sensitive factor that reflects vascular indicators (Sun et al., 2020), cardiac contractility, blood viscosity, and the position of the Doppler probe interfere with the results, which may be the reason for the discrepancy between these studies. Therefore, the use of Doppler to evaluate the effect of ephedrine has some flaw. A recent study by Shapiro (Shapiro et al., 2020), used blood oxygen level-dependent magnetic resonance imaging (BOLD-MRI), which closely reflects oxygen delivery or extraction and has been used to accurately image the hypoxic uterus, to compare the effects of ephedrine administration on placental circulation (Wedegärtner et al., 2010). This study showed that 10–20 mg ephedrine increases the oxygen supply to the placenta, which is closely related to uterine artery dilatation. This is consistent with our conclusion that ephedrine dilates the uterine artery at this dose.

\However, there are several limitations to our study. First, the study was based on one animal models. The animal model is characterized by superficial trophoblasts on the surface of the uterine decidua, which makes the uterine artery sensitive to the sympathetic nerve (Adamson et al., 2002; Carter, 2020). In contrast, in the human womb, trophoblasts are present deep within the uterine myometrium (Brosens et al., 2002). This causes subtle changes in the uterine artery, which may change the sensitivity to vasoconstrictor drugs or sympathetic nerves (Hamzic et al., 2008; Osol GandMandala, 2009). Moreover, the study was based on the uterine artery, and we have not discussed blood vessels, such as the placental arcuate artery and fetal umbilical cord, which are closely related to fetal health. Further studies are required to explore the effect of different doses of ephedrine in the uteroplacental circulation of pregnant women, and the differences in anesthesia methods also need further research.

In summary, CT can be used to non-invasively evaluate the changes in peripheral blood vessels over a short period of time. Additionally, this study showed that the peripheral artery contracts under the action of ephedrine, whereas the common clinical dose of ephedrine has no significant effect on the diameter of the uterine artery. However, at 30 mg, ephedrine can significantly inhibit the diameter of the uterine artery.

## Data Availability

The raw data supporting the conclusions of this article will be made available by the authors, without undue reservation.
